# Using a structured questionnaire improves seizure description by medical students

**DOI:** 10.5116/ijme.566c.096c

**Published:** 2016-01-10

**Authors:** Saher Kapadia, Hemang Shah, Nancy McNair, J. Ned Pruitt, Anthony Murro, Yong Park

**Affiliations:** 1 Medical College of Georgia, Georgia Regents University, Augusta, Georgia, 30912, USA; 2 Neurology Department, Medical College of Georgia, Georgia Regents University. Augusta, Georgia, 30912, USA

**Keywords:** Seizure description, seizure questionnaire, seizure characteristics, seizure semiology

## Abstract

**Objectives:**

The purpose of this study was to evaluate a structured questionnaire for improving a medical students’ ability to identify, describe and interpret a witnessed seizure.

**Methods:**

Ninety two 3rd year medical students, blinded to seizure diagnosis, viewed videos of a primary generalized seizure and a complex partial seizure.  Students next completed an unstructured questionnaire that asked the students to describe the seizure video recordings. The students then completed a structured questionnaire that asked the student to respond to 17 questions regarding specific features occurring during the seizures.  We determined the number and types of correct responses for each questionnaire.

**Results:**

Overall, the structured questionnaire was more effective in eliciting an average of 9.25 correct responses compared to the unstructured questionnaire eliciting an average of 5.30 correct responses (p < 0.001). Additionally, 10 of the 17 seizure features were identified more effectively with the structured questionnaire. Potentially confounding factors, prior knowledge of someone with epilepsy or a prior experience of viewing a seizure, did not predict the student’s ability to correctly identify any of the 17 features.

**Conclusions:**

A structured questionnaire significantly improves a medical student’s ability to provide an accurate clinical description of primary generalized and complex partial witnessed seizures. Our analysis identified the 10 specific features improved by using the structured questionnaire.

## Introduction

Seizures are common, affecting about 4% of the population by age 80, and reoccurring in about 30-40% of those with a first seizure.^1^ Of greater concern, misdiagnosis is common; about 30-40% of patients evaluated at epilepsy centers are not epileptic.^2^ Accurate seizure identification and classification requires an accurate account of the behavioral events occurring during the seizure (seizure semiology). This account of behavioral events contributes significantly to identifying the seizure onset site.^3^ A detailed account of behavioral events also contributes to improved differential diagnosis of non-epileptic events from epileptic events, selection of patients for clinical trials, and selection of patients for epilepsy surgery.^4^

Accurate seizure identification and classification remains a challenging task because of the complexity of seizure behavior and the limited accuracy of witnessed reports.  The International League Against Epilepsy (ILAE) Glossary exemplifies the complexity of seizure behaviors, listing 91 distinct terms for characterizing seizure semiology.^5^  A twin questionnaire study exemplified the limited accuracy of seizure reports; in one subpopulation, 18.5% of participants with verified epilepsy reported no prior history of seizures.^6^  In a study of eyewitness reports of seizure video recordings, eyewitnesses often overlook or inaccurately recall salient features of seizures.^7^  Even among clinicians, seizure reports obtained from the same patient by different clinicians from may vary.^8^ In a study of children with a first unprovoked seizure, clinicians had only a moderate to high level of agreement for only 68% of the 31 clinical variables studied.^8^  Similarly, medical students often fail to distinguish psychogenic non-epileptic seizures from epileptic seizures.^9^  Varying interpretations of witnessed reports also lead to inconsistent seizure classification. Among trained lay reviewers, agreement of seizure diagnosis varied significantly.^10^ In a study comparing initial to final diagnosis, 22% of first seizure cases were initially incorrectly classified as non-epileptic events by accident and emergency department physicians.^11^

The challenge of accurate seizure diagnosis has led to research in epilepsy education.  Isler et al. developed an effective modular epilepsy educational program for medical residents, nurses and EEG technicians.^12^ Using a randomized crossover trial of epilepsy education, Bye et al. found that although interactive lecturing and a computerized tutorial were both effective, students preferred interactive learning^13^.  Bye et al. also evaluated medical students’ interest, clinical confidence and perceived usefulness of their epilepsy educational program.^13^

The focus of this study was to develop an educational resource that would improve a student's ability to interpret a witnessed seizure.  Our hypothesis was that a structured questionnaire would guide a student's recall, improving the student's ability to identify, describe and interpret witnessed seizures.   

## Methods

### Setting and participants

The Georgia Regents University Institutional Review Board provided ethical approval for this research.  This study recruited 3rd year medical students who were enrolled in the Georgia Regents University Neurology Clerkship over a 1 year interval.  This study was in compliance with ethical regulations at Georgia Regents University.

### Seizure observation and questionnaire completion

During the medical student’s second year, all students received a 1 hour epilepsy lecture that included video recordings of complex partial and primary generalized seizures.  During the first day of their 3rd year neurology clerkship, students were asked to view 2 videos.  These students were not told that the videos recorded seizures or that they would receive a questionnaire.  The first video recorded an adult male with a 43 second primary generalized tonic-clonic seizure (juvenile myoclonic epilepsy) with bilateral myoclonus and ictal vocalization.  The second video recorded an adult female with a 63 second complex partial seizure (temporal lobe epilepsy) with pause of activity, oral automatisms and manual automatisms.  The seizures occurred in alert patients, lying in bed with scalp electrodes in an inpatient epilepsy monitoring unit.  The patients provided written consent for viewing of the video recordings. 

After viewing both videos, the students first completed the unstructured questionnaire which contained 2 questions: Please describe what you see in video 1, please describe what you see in video 2. 

After completing the unstructured questionnaire, the students completed the structured questionnaire. The students identified their gender, reported if they had seen a seizure previously, and reported if they knew someone who had a seizure disorder. The structured questionnaire contained 17 questions, evaluating 8 features from the primary generalized seizure and 9 features from the complex partial seizure.  The structured questionnaire asked the student if the event was a seizure, how did the seizure begin, how long did the seizure last, which limb jerked first, did an automatism occur, did any vocal activity occur, did the seizure appear to rise from the right or left side of the brain, and did the patient appear to lose consciousness; for the complex partial seizure, the questionnaire asked if the head turned to the right or left side. Seizure duration was considered correct if the time estimate agreed to within +15 seconds of the actual seizure duration.  We selected complex partial and primary generalized seizures and the specific seizure features because these seizure types and seizure features are commonly encountered in clinical practice.  We evaluated these 17 features because our educational objective was for the students to identify, describe and interpret complex partial and primary generalized witnessed seizures.

### Data analysis

The first outcome measurement was the total number of correctly identified seizure features using the unstructured questionnaire and total number of correctly identified features using the structured questionnaire.  We determine this outcome measure for each student. We applied the paired student t test to this data to determine if the structured questionnaire significantly improved the total of number of correctly identified features. Using a linear regression model, we determined if two potentially confounding variables, prior experience of seeing a seizure and knowing someone with a seizure disorder, could predict the change in total number of correctly identified items from using the structured questionnaire.

The second outcome measure was a binary variable representing either improved or worsened ability to identify a specific seizure feature.  Correct identification of the seizure feature on the structured questionnaire and incorrect identification on the unstructured questionnaire represented an improved outcome.  Incorrect identification of a seizure feature on the structured questionnaire and correct identification on the unstructured questionnaire represented worsened outcome. We determined this outcome measure for each student and for each of the 17 seizure features.  We used the sign test to determine if the improved outcomes were more likely than worsened outcomes for each individual seizure feature.  Using a logistic regression model, we determined if two potentially confounding variables, prior experience of seeing a seizure and knowing someone with a seizure disorder could predict, improved or worsened outcome for each seizure feature.  We used a p < 0.0029 for statistical significance which corresponds to familywise p < 0.05 adjusted for 17 repeated measurements.  We performed statistical analysis using R statistical software and the mid-p value method.^14^^,^^15^

## Results

This study enrolled 92 participants, 35 (38%) women and 57 (62%) men.  An additional 2 participants initially participated but were later excluded because they did not complete the questionnaire. 

The overall number of participants who correctly identified each feature varied over a wide range (Table 1). Overall, the structured questionnaire was more effective in eliciting an average of 9.25 correct responses compared to the unstructured questionnaire eliciting an average of 5.30 correct responses (p < 0.001).  The 2 potential confounding factors, prior experience of seeing a seizure and knowing someone with a seizure disorder, were not statistically significant predictors of the total number of correct responses from using a structured questionnaire.

**Table 1:** Number of participants identifying each feature correctly (N=92)

Seizures		Structured questionnaire Frequency (%)	Unstructured questionnaire Frequency (%)
Primary generalized Seizure			
	Recognize seizure	85 (92)	62 (67)
	Limb jerking at onset	66 (72%)	57 (62)
	Duration 28-58 seconds	0 (0)	9 (10)
	Bilateral arm jerking	42 (46)	52 (57)
	No automatisms	56 (61)	0 (0)
	Ictal vocalization	80 (87)	46 (50)
	Bilateral cerebral onset	54 (59)	45 (49)
	Consciousness impaired	29 (32)	9 (10)
Complex partial seizure			
	Recognize seizure	56 (61)	15 (16)
	Pause of motor activity	69 (75)	27 (29)
	Duration 48-78 seconds	15 (16)	3 (3)
	Right arm jerking first	69 (75)	64 (70)
	Automatisms present	90 (98)	83 (90)
	No head or eye deviation	37 (40)	1 (1)
	Ictal vocalization absent	78 (85)	3 (3)
	Right cerebral onset	8 (9)	0 (0)
	Consciousness impaired	22 (24)	4 (4)

The relative improvement from using a structured questionnaire was significant for 10 of the 17 features using a familywise p-value 0.05 adjusted for 17 repeated measures (Table 2).   For both seizures, the structured questionnaire improved the student's recognition of seizure occurrence, absence or presence of ictal vocalization, and impaired consciousness (Table 2).  The structured questionnaire also improved student recognition for absence of automatisms for the primary generalized seizure and pause of motor activity, duration, and absence of head or eye deviation for the complex partial seizure (Table 2).  The 2 potential confounding factors were not statistically significant for predicting the improved performance from using a structured questionnaire for any of the individual 17 features (Table 3).

## Discussion

In comparison to prior research, our study was unique in verifying that a specific intervention, a study questionnaire, more effectively elicited the major features of an observed seizure compared to an unstructured questionnaire.  This benefit occurs for distinct seizure types, complex partial and primary generalized seizures. Also, important is that structured questionnaire improves recognition of some but not all seizure features and prior experience of seeing a seizure or knowing someone with seizures does not improve the student’s ability to recognize seizure features.

**Table 2:** Number of participants who improved or worsened using a structured questionnaire (N=92)

Seizures		Improved^†^ Frequency (%)	Worsened ^‡^ Frequency (%)
Primary generalized seizure			
	Recognize seizure*	24 (26)	1 (1)
	Limb jerking at onset	25 (27)	16 (17)
	Duration 28-58 seconds	0 (0)	9 (10)
	Bilateral arm jerking	17 (18)	27 (29)
	No automatisms*	56 (61)	0 (0)
	Ictal vocalization*	34 (37)	0 (0)
	Bilateral cerebral onset	25 (27)	16 (17)
	Consciousness impaired*	23 (25)	3 (3)
Complex partial seizure			
	Recognize seizure*	44 (48)	3 (3)
	Pause of motor activity*	43 (47)	1 (1)
	Duration 48-78 seconds*	13 (14)	1 (1)
	Right arm jerking first	14 (15)	9 (10)
	Automatisms present	8 (9)	1 (1)
	No head or eye deviation*	36 (39)	0 (0)
	Ictal vocalization absent*	75 (82)	0 (0)
	Right cerebral onset	8 (9)	0 (0)
	Consciousness impaired*	21 (23)	3 (3)

*p< 0.0029 corresponds to familywise p< 0.05 adjusted for 17 repeated measurements. †Improved: Structured questionnaire response correct, unstructured questionnaire response incorrect. ‡Worsened: Structured questionnaire response incorrect, unstructured questionnaire response correct.

There are some limitations of this study. This study’s questionnaire identified the major seizure features in the 2 videos viewed by the students. However, a single standard questionnaire surveying the full range of recognized behavioral features could apply to a broader range of seizure types.^5^ In this study, our selection of a limited number of questionnaire items could cause potential bias.  A comprehensive standard seizure questionnaire could reduce this potential for bias.  The unstructured questionnaire asked the students to describe what they saw but did not specifically request a detailed description; a request for a detailed description may have led to a greater number of correct responses on the unstructured questionnaire. This study relied on 2 seizures; however, variability occurs among seizures and use of a greater number of seizures would generate a more accurate estimate of which seizure features are likely to be recognized by medical students.

In this study, we restricted the study to complex partial and primary generalized seizures, but for medical student instruction, video instruction would need to include a broad range of seizure disorders, syncope and non-epileptic psychogenic seizures. Our data shows that the combined sequence of unstructured and structured questionnaire is more effective than unstructured questionnaire alone.  Due to the study design, we cannot conclude that structured questionnaire alone is better than unstructured questionnaire alone. Although the structured questionnaire improved student performance, the mean score of 9.35 items correct was below the ideal score of all 17 items correctly identified. Another limitation of this study was that although we identified which features were improved with the structured questionnaire, our data did not indicate why performance improved on only 10 of the 17 seizures features.

**Table 3:** Logistic regression coefficients for potential confounding factors

Seizures		Prior knowledge of someone with epilepsy	Prior experience of viewing a seizure
		Regression coefficient (p value)	Regression coefficient (p value)
Primary generalized seizure			
	Recognize seizure	0.0000 (1.0000)	16.5703 (0.9983)
	Limb jerking at onset	-0.7339 (0.3055)	0.8072 (0.3068)
	Duration 28-58 seconds	0.0000 (1.0000)	0.0000 (1.0000)
	Bilateral arm jerking	-0.1889 (0.8031)	1.4005 (0.0564)
	No automatisms	0.0000 (1.0000)	0.0000 (1.0000)
	Ictal vocalization	0.0000 (1.0000)	0.0000 (1.0000)
	Bilateral cerebral onset	-0.2009 (0.8124)	-0.0732 (0.9221)
	Consciousness impaired	-23.3136 (0.9982)	22.0288 (0.9988)
Complex partial seizure			
	Recognize seizure	-0.8238 (0.5230)	-0.5487 (0.6692)
	Pause of motor activity	19.2772 (0.9987)	-20.3565 (0.9980)
	Duration 48-78 seconds	18.6201 (0.9988)	18.6202 (0.9983)
	Right arm jerking first	-0.4821 (0.6499)	0.5797 (0.6125)
	Automatisms present	-49.5282 (0.9997)	48.5251 (0.9998)
	No head or eye deviation	0.0000 (1.0000)	0.0000 (1.0000)
	Ictal vocalization absent	0.0000 (1.0000)	0.0000 (1.0000)
	Right cerebral onset	0.0000 (1.0000)	-17.8729 (0.9969)
	Consciousness impaired	0.0549 (0.9669)	-0.7584 (0.5742)

Two prior studies also investigated diagnostic accuracy of seizure classification using video recorded seizures.  Using a logistic regression model, Azar et al. constructed a model based on a 12 item questionnaire that would distinguish epileptic from non-epileptic seizures with a predicted 84.4% accuracy.^16^ Unlike our study, Azar et al. did not determine the relative benefit of their questionnaire in a controlled trial.^16^ Hirfanoglu et al. compared seizure classification before and after viewing seizure video recordings.^17^  Hirfanoglu et al. found a moderate to high consistency of seizure classification for 6 of the 22 seizure types.^17^ Unlike our study, Hirfanoglu et al. investigated a far larger number of seizure types, but did not investigate identification of specific seizure features.^17^ Also unlike our study, Hirfanoglu et al. investigated the influence of a different manipulated variable, seizure video data, on seizure classification.^17^ In comparison to the prior studies, less experienced medical students in our study observed and interpreted seizure behavior rather than the more experienced physicians in these prior studies.^16^^,^^17^

The results of this study may lead to additional research and new applications in the future.  Future research could determine if repeated application of the unstructured and structured questionnaire sequence would train students to consistently perform better, ultimately recognizing all features of a video recorded event.  Future research could focus on the effects of structured questionnaires on recognizing key features of other clinical events by participants other than medical students: the lay person, non-physician professionals, primary care physicians and neurologists.  Questionnaires could be developed to evaluate related disorders such as psychogenic nonepileptic seizures, convulsive syncope and other seizure types. The questionnaire could be applied prior to and following epilepsy student lecture to determine if the lecture was effective in promoting student learning. Another approach would be for a neurologist, blinded to seizure diagnosis, to diagnose the seizure type from the unstructured student questionnaire responses; the features from the unstructured questionnaires leading to correct diagnosis would identify the features sufficient for diagnosis.  A seizure questionnaire, modified for use by a layperson, may be useful to evaluate responses to anti-epileptic medications, investigational medications or vagus nerve stimulator therapy in the home environment.

## Conclusions

This study shows that a structured questionnaire significantly improves a medical student’s ability to accurately identify, describe and interpret the key features of primary generalized and complex partial witnessed seizures.  This study identified 10 specific features improved by using the structured questionnaire. 

### Acknowledgements

We gratefully acknowledge the participation of all the students involved in this study. We also acknowledge the patients who consented to the viewing of video recordings of their seizures.

### Conflict of Interest

The authors declare that they have no conflict of interest.
